# Histology-informed automatic parcellation of white matter tracts in the rat spinal cord

**DOI:** 10.3389/fnana.2022.960475

**Published:** 2022-11-29

**Authors:** Harris Nami, Christian S. Perone, Julien Cohen-Adad

**Affiliations:** ^1^NeuroPoly Lab, Institute of Biomedical Engineering, Polytechnique Montréal, Montreal, QC, Canada; ^2^Functional Neuroimaging Unit, CRIUGM, Université de Montréal, Montreal, QC, Canada; ^3^Mila – Quebec AI Institute, Montreal, QC, Canada

**Keywords:** microscopy, histology, clustering, machine learning, spinal cord, white matter, rat

## Abstract

The white matter is organized into “tracts” or “bundles,” which connect different parts of the central nervous system. Knowing where these tracts are located in each individual is important for understanding the cause of potential sensorial, motor or cognitive deficits and for developing appropriate treatments. Traditionally, tracts are found using tracer injection, which is a difficult, slow and poorly scalable technique. However, axon populations from a given tract exhibit specific characteristics in terms of morphometrics and myelination. Hence, the delineation of tracts could, in principle, be done based on their morphometry. The objective of this study was to generate automatic parcellation of the rat spinal white matter tracts using the manifold information from scanning electron microscopy images of the entire spinal cord. The axon morphometrics (axon density, axon diameter, myelin thickness and g-ratio) were computed pixelwise following automatic axon segmentation using AxonSeg. The parcel**l**ation was based on an agglomerative clustering algorithm to group the tracts. Results show that axon morphometrics provide sufficient information to automatically identify some white matter tracts in the spinal cord, however, not all tracts were correctly identified. Future developments of microstructure quantitative MRI even bring hope for a personalized clustering of white matter tracts in each individual patient. The generated atlas and the associated code can be found at https://github.com/neuropoly/tract-clustering.

## Introduction

The spinal cord is part of the central nervous system, and one of its purposes is to ensure communication between the peripheral nervous system and the brain. This communication happens along axons, which are organized into “tracts,” “bundles” or “funiculi” and form the white matter. Some pathologies, such as multiple sclerosis and spinal cord injury, yield to the degradation of white matter axons and consequently their ability to send electric signaling. This degradation in communication between the brain and the peripheral nervous system can produce devastating motor and/or sensory deficits. In this context, researchers like to better understand the anatomy and physiology of the spinal cord, notably the cyto- and myeloarchitecture of the various white matter tracts. These tracts are defined based on which neuronal population they originate from and synapse to. Each tract is consequently associated with a particular function. For example, the corticospinal tract, which is a descending tract (i.e.,: the neuronal body is located in the supratentorial brain and the action potential descends down the spinal cord), commands muscles to execute motor functions. Hence, a degradation of the corticospinal tract as seen in amyotrophic lateral sclerosis patients, will lead to a loss of motor functions. It is therefore relevant, from a diagnosis but also from a drug development standpoint, to know where these tracts are located.

Traditionally, the tract location is obtained by staining specific neuron populations: the contrast agent diffuses along the associated axons, and the boundary of the white matter tracts is visualized via optic microscopy and then documented on a manual sketch. When atlases of white matter tracts are built from several specimens of a given species, this tedious process is repeated, and then results are qualitatively interpreted to produce a spatially averaged map of the various tracts in that given species. In addition to issues related to the staining procedure – sometimes the contrast agent would diffuse to other tracts–, these methods are extremely time consuming and biased by the operator interpreting the microscopy scans and doing the manual sketching. For these reasons, only scarce resources are available on the precise location of white matter tracts (Saliani et al., 2017). A popular white matter atlas for humans is the eponym “Gray’s anatomy” atlas (Standring, 2008), and an equivalent for rats would be the “Watson” atlas (Gulgun et al., 2015). While daunting work was put in to generate these precious resources, these atlases are not fully comprehensive and are built from only a handful of specimens. For example, in the Watson rat atlas not all the tracts were seen by the staining techniques (in comparison with the mouse atlas from the same authors), hence some tracts are missing in the atlas.

Interestingly, axons from a given tract exhibit specific characteristics in terms of morphometrics and myelination. For example, axons in the cuneatus (ascending tract in the dorsal column) are known to be smaller and denser than that in the spinocerebellar tracts (Duval et al., 2019). Hence, one could wonder if the parcellation of the white matter tract could be done (partly or entirely) based on their morphometry? This hypothesis has already been investigated, notably by Assaf et al. (2008), who used diffusion-weighted magnetic resonance imaging to quantify the size and density of axons in the porcine spinal cord. This voxel-wise mapping was followed by a k-means clustering to regroup voxels exhibiting similar diffusion-related metrics (and, indirectly, similar axon morphometrics). The resulting clustering was convincingly close to the known delineation of the white matter tracts based on traditional staining techniques. Given the recent improvement on large-throughput mapping of axon morphometrics in the spinal cord using electron microscopy (Duval et al., 2019; Saliani et al., 2019), one could wonder if automatic clustering from these high resolution microscopy scans, aggregated across multiple specimen, would produce reliable clusters that mimic the known distribution of white matter tracts.

The objective of this study was to generate automatic parcellation of the rat spinal white matter tracts using the manifold information from scanning electron microscopy images of the entire spinal cord (C1 to S4) obtained in 5 rats. The axon morphometrics (axon density, axon diameter, myelin thickness and g-ratio) were computed pixelwise following automatic axon segmentation using AxonSeg (Zaimi et al., 2016). The parcellation was based on an agglomerative clustering algorithm to group the tracts. The generated atlas and the associated code to be able to fully reproduce the atlas can be found at https://github.com/neuropoly/tract-clustering.

## Materials and methods

This study is a follow-up of a previous publication that described the construction of a rat spinal cord atlas of axon morphometry (Saliani et al., 2019). The animal/tissue preparation, scanning and atlas creation steps are briefly described in the first section below, but for more details the reader is referred to the previous publication. The subsequent sections focus on the histology-informed clustering of spinal pathways.

### Microscopy atlas creation

#### Animal preparation

All experimental protocols were carried out according to the guide- lines of the Canadian Council on Animal Care regarding the care and use of animals for experimental procedures. The protocols were approved by the Animal Research Ethics Committee of the Montreal Heart Institute. Every attempt was made to minimize animal suffering and to reduce the number of rats used.

The spinal cords of 5 sprague-dawley rats were used for this study. Rats were anesthetized by perfusion and fixed with a mixture of 3% paraformaldehyde and 3% glutaraldehyde. Upon extraction, the spinal cords were post-fixed in the same fixative solution and then cut into the separate 31 spinal cord levels, from C1 to S4. These were then dehydrated in varying concentrations of acetone baths and then stained with 2% osmium tetroxide (for myelin imaging). They were then embedded into an epon resin so that they could be adequately prepared for imaging.

#### Microscopy imaging

The samples embedded in the resin were polished to obtain a smooth surface finish of the area to be imaged (final grit of 0.05 μm). The samples were coated with gold to improve image contrast. Imaging was performed with a scanning electron microscope (JEOL JSM7600F) at a 130 nm resolution. Each spinal level was imaged as multiple sub-images of 8192 × 5632 pixels (the number of sub-images varied based on sample size) and then stitched together at the end using in-house MATLAB scripts and the Fiji software to obtain the whole image of the level. This was done for all 31 levels of the rat spinal cord. The quality of tissue preparation and imaging can be appreciated in Supplementary Figure 1.

#### Segmentation of axons and myelin

Stitched images were processed with AxonSeg (Zaimi et al., 2016) to generate masks of individual axons and myelin. Metric maps of axon density, axon diameter, axon volume fraction, g-ratio and myelin thickness were obtained from the segmentation. These maps were generated at a 50 × 50 μm resolution. Each pixel of this map represents the average value of a given metric within the pixel, except for the axon volume fraction, which corresponds to the axon count per pixel unit surface.

#### Creation of axon morphometry template

White matter binary masks were created for each slice and each rat in the downsampled space. Based on these masks, affine and bsplineSyN transformations (Tustison and Avants, 2013) were computed to co-register all rats together, on a slice-by-slice basis, imposing right-left symmetry. The transformations were then applied to the metric maps and these were averaged across rats to obtain an average template of all the axon/myelin metrics.

#### Registration of Watson spinal cord atlas

The Watson et al. (2009) white matter atlas of the rat spinal cord was semi-manually digitized into discrete labels (one tract corresponding to a label) and right-left symmetrized. A binary white matter mask of the atlas was generated and then used to estimate an affine and non-linear (bsplineSyN) transformation between the Watson atlas and the axon morphometry template generated above. This transformation was then applied to individual labels in order to obtain Watson’s white matter tracts in the space of the axon morphometric template. These tracts were subsequently used as reference to validate the quality of obtaining these tracts with a data-driven approach (i.e.,: from the axon morphometric maps).

**FIGURE 1:**
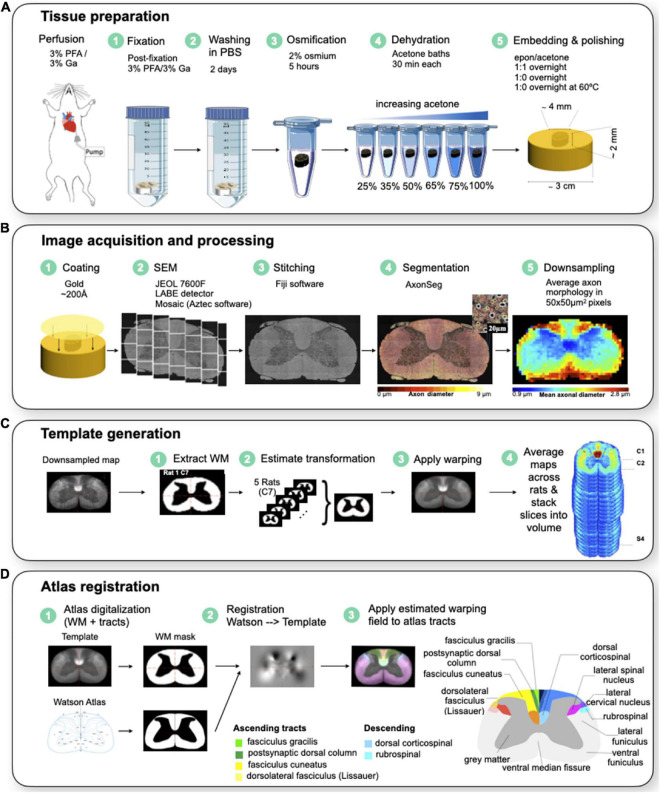
**(A)** Animal/tissue preparation. The rat is first perfused and fixed to extract the spinal cord. The spinal cord is fixed in a 3% PFA and 3% GA solution for 2 days and washed in PBS for a subsequent 2 days before being stained with 2% Osmium tetroxide for 5 h. The cords are dehydrated in varying acetone baths before being embedded in a resin at 60 celsius overnight. The samples are polished to a 0.05 um surface finish. **(B)** Image acquisition and processing. The samples are imaged using a scanning electron microscope at 200X magnification for a 130 nm resolution. The images are stitched together using Fiji with an in-house MATLAB script to obtain a high resolution image of the spinal cord. **(C)** Template generation. A binary WM mask was created from downsampled maps for each sample, and put in a common space. The mask was then centered and aligned in the same direction. Affine and BsplineSyn transformations were used to register samples. The template was generated at each level, and was then concatenated into a single 4D NIfTI data structure. **(D)** Atlas registration. The Watson et al. spinal cord white matter atlas was symmetrized. A binary WM mask was made for the Watson et al. atlas and the template. The atlas registration to template was estimated based on the masks of white and gray matter of the atlas (source) and generated template (destination). WM tracts were registered by applying the estimated warping fields to the atlas tracts. WM tracts were then put in the same space as the template using the transformation. Modified with permission from Saliani et al. (2019).

Figure 1 illustrates the workflow for animal/tissue preparation and imaging.

**FIGURE 2:**
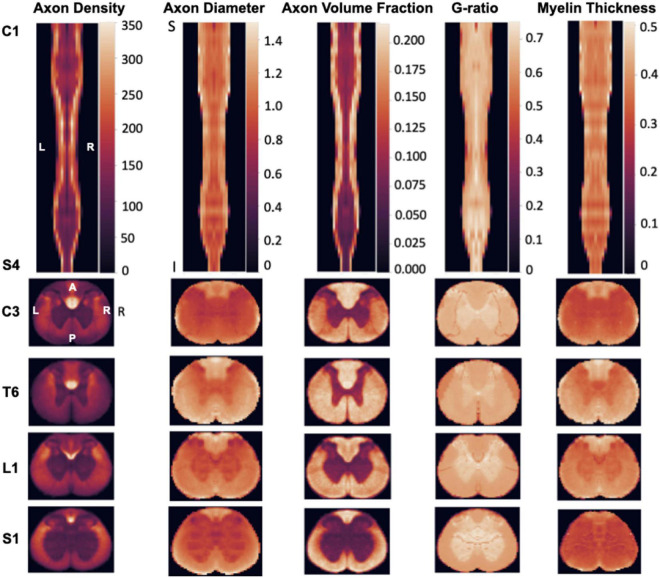
Morphometric maps from the rat spinal cord template (*N* = 5 rats). **(Top Row)** Coronal view metric map covering the entire spinal cord (from C1 to S4). **(Bottom Rows)** Selected axial views from each section of the spinal cord (Cervical, Thoracic, Lumbar, Sacral). Note that the scale of each axial view was adjusted to fit the rectangle panel, so the relative size of the spinal cord across levels cannot be compared across axial views.

Figure 2 illustrates the generated morphometric template, which was inputted into the clustering algorithm (described in the next section). The maps show a variation in the spread of metrics based on the region and tract in the spinal cord. For instance, the average axon density ranged from 78,000 axons/mm^2^ in the fasciculus gracilis to 168,800 axons/mm^2^ in the dorsal corticospinal tract. The mean axon diameter ranged from 1.1 in the dorsal corticospinal tract to 1.35 in the dorsal column. The myelin thickness ranged between 0.35 and 0.5 μm, and the myelin volume fraction ranged between 15 and 30%. We hypothesized that this tract-specific ‘morphometric signature’ would produce a robust data-driven clustering that would spatially delineate individual tracts, as described hereafter.

### Slice-wise clustering

A first (and intuitive) attempt for the data-driven clustering of spinal tracts consisted in inputting a 2D axial slice (corresponding to one spinal level) for each of the five morphometrics. Hence 31 clusterings were performed independently (one for each spinal level).

Only voxels inside the white matter mask were considered for the clustering. Each morphometric map was normalized between 0 and 1 before entering the clustering.

Clustering of the morphometrics data was performed using scikit-learn’s agglomerative clustering algorithm. The number of clusters was varied between 5 and 11 (with an increment of 1) to explore the impact of this parameter on the generated clusters. A connectivity matrix was defined to enforce pixel-to-pixel connection within the axial plane. Clusters were merged by minimizing the variance of the Euclidean distance between cluster candidates (linkage = ward, affinity = Euclidean).

### Region-wise clustering

While the slice-wise approach described above seemed the most faithful to the data, it produced unsatisfactory results (see results section), which we attributed to noise and to the relatively low number of samples. To obtain a more robust clustering, we enforced additional spatial priors, namely: we aggregated slices within each of the four spinal regions (cervical, thoracic, lumbar and sacral), and we non-linearly co-registered slices together within the same region to be able to define the connectivity matrix along the superior-inferior axis in addition to the axial plane. The approach is described hereafter.

**FIGURE 3:**
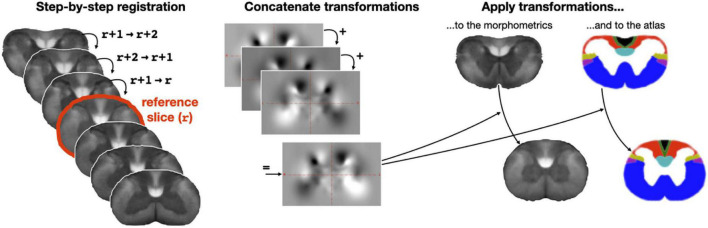
Step-by-step slice-wise registration. Slices from the morphometric template are non-linearly registered to their adjacent slice until reaching a reference slice, which is defined as the mid-slice within a region (here the cervical region is shown). Then, transformations are concatenated in a step-by-step fashion and the transformation corresponding to a given slice is applied to each morphometric map and to the Watson atlas. This procedure is repeated for each region (cervical, thoracic, lumbar, sacral).

Building on the anatomical knowledge that the spatial distribution of tracts is relatively similar within a given region (e.g., cervical, thoracic), we ran the clustering by aggregating adjacent axial slices within a given region. But before doing that, to account for the slight displacement of funiculi across slices, we co-registered each axial slice to the slice located at the center of a region. This co-registration was performed using ANTs’ bspline-regularized SyN transformation on the axon density metric map. To ensure a smooth and robust transformation, instead of directly registering each slice to the center slice, we instead performed a step-by-step registration where the center-slice *r* is registered with slice *r* + 1, then slice *r* + 1 is registered with slice *r* + 2, and so on. Transformations were then concatenated and applied to the corresponding slice. This algorithm is illustrated in Figure 3. Following co-registration, morphometric data were averaged along the superior-inferior axis, producing 2D slices for each region (*n* = 4) and each morphometric (*n* = 5), so 20 in total.

For convenient comparison with the Watson atlas, the estimated warping fields were also applied to the digitized Watson atlas. Then, as done for the morphometrics data, the region-specific registered Watson slices were averaged together, resulting in a single 2D axial slice per region. Technical note: each tract was defined in a 4th dimension, so that averaging across the 3rd dimension did not result in an “overlap” of the tracts, i.e., each z-averaged tract ranged from 0 to 1 in order to estimate partial volume information, which was subsequently used to produce colored maps of the atlas for qualitative assessment.

### Validation

A quantitative validation of the clustering results was tricky to implement because there was no clear way to justify what cluster to consider for computing a distance (e.g., Hausdorff) or overlap (e.g., Dice) metric between the clusters and tracts from the Watson atlas. Therefore, we opted for a qualitative (visual) evaluation, as done in Assaf et al. (2008). Moreover, the qualitative evaluation was augmented by quantitatively selected colormaps and intensity: we generated 2D images showing the clusters next to the Watson atlas, with matching color-coding based on the level of overlap. When multiple clusters were found within a given tract, the intensity level would be varied between 0.2 and 1, scaled with the amount of overlap with the Watson tract.

## Results

### Slice-wise clustering

**FIGURE 4:**
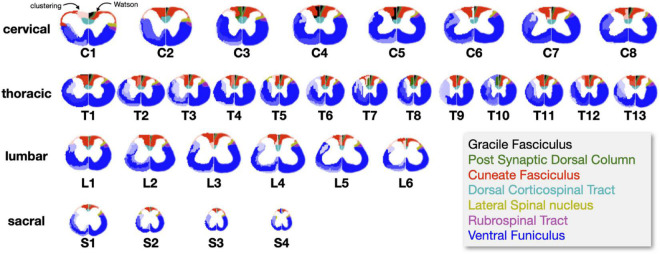
Results of the slice-wise clustering for each spinal level (set to 8 clusters). Clustering results are shown on the left side of each spinal cord, and the Watson atlas is shown on the right side. Colors were chosen to topologically match between the two sides for easier assessment. Wherever the clustering resulted in parcellated clusters (where the Watson atlas would only show one funiculi), the intensity across parcellated clusters was varied (e.g., light blue, dark blue). The spatial scaling is kept the same.

Figure 4 shows, for each spinal level, the clustering results (left hemi-section) and the Watson atlas (right hemi-section). The color coding was topologically matched for convenient comparison. Here, the number of clusters was set to 8, which corresponds to the number of tracts in the Watson atlas. We observe a somewhat good topological correspondence between the generated clusters and that from the Watson atlas, notably for the cuneate fasciculus (red) and the dorsal corticospinal tract (cyan). Some smaller tracts (gracile, post synaptic dorsal column, lateral spinal nucleus, rubrospinal) are not well captured by the clustering. The ventral funiculus, which is a large collection of ventral tracts from the Watson atlas, appears parcellated on the generated clusters, and this parcellation is not consistent across slices. This lack of consistency is possibly related to noise from the histology data and to the ill-posed problem of clustering.

### Region-wise clustering

**FIGURE 5:**
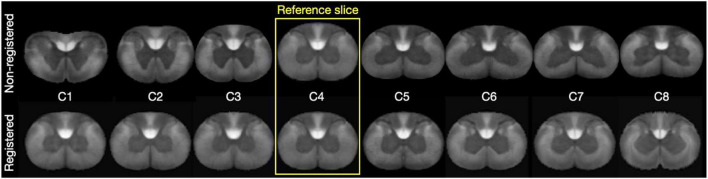
**(Top Row)** Shows the individual slices for the cervical level for the metric map “Axon density”. The **(Bottom Row)** shows the registered levels. C4 was the reference slice for registration.

The inconsistencies observed with the slice-wise clustering were tackled by aggregating slices within the same region (cervical, thoracic, lumbar, sacral). To achieve this, we first non-linearly registered slices pertaining to the same group. Figure 5 shows satisfactory results of this inter-slice registration for the cervical region. Registration went equally well for the other three regions.

**FIGURE 6:**
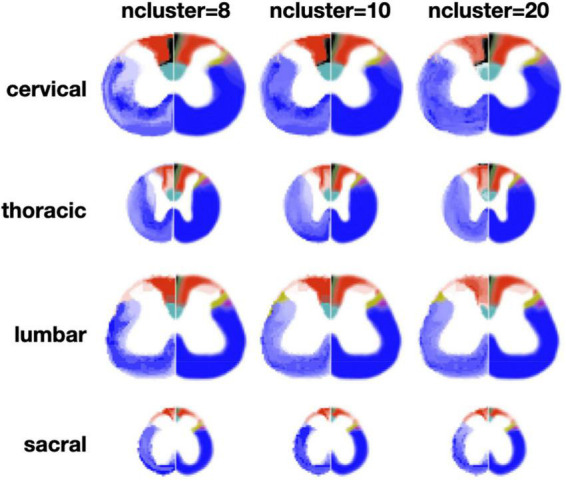
Results of the region-wise clustering. Clusters are shown for 8, 10, and 20 clusters for each region after they have been registered to a reference slice within each region. The left half shows the clustering results and the right half shows the Watson atlas. The spatial scaling across regions is kept the same.

The clustering algorithm was applied using the registered slices as inputs for all the metric maps. Figure 6 shows results of the clustering for *n* = 8 and *n* = 10 clusters. Like for the slice-wise clustering, the generated clusters are shown on the left section of the spinal cord while the Watson atlas is shown on the right section. Overall, the same two clusters (cuneate fasciculus, dorsal corticospinal tract) are clearly delineated across all four regions, with a particularly good topological correspondence for the cervical and thoracic regions. The Gracile Fasciculus (black) and the Post Synaptic Dorsal Column (green) appear to be merged into the same cluster (light red) for the cervical and thoracic regions. This merging could be caused by similar morphometrics features between these two tracts, that the automatic clustering was not able to distinguish. The sacral slice seems a bit “noisy,” which could be due to the fact that tracts are smaller in the lower spinal cord region.

**FIGURE 7:**
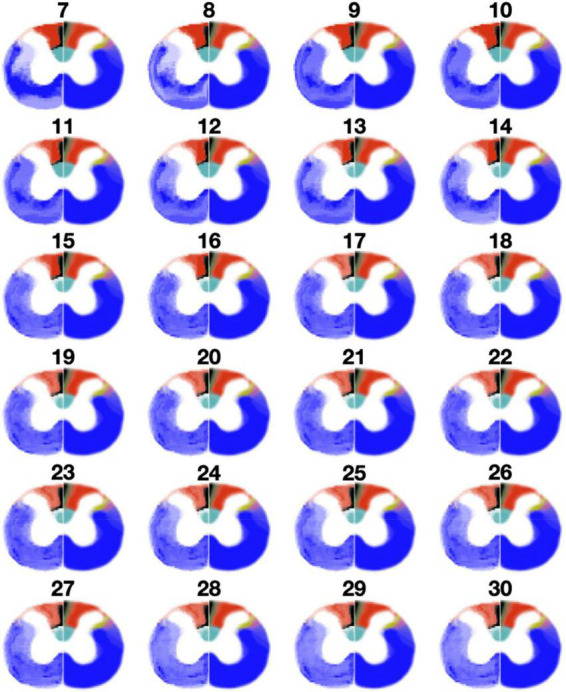
Results of clustering with a varying number of clusters (from 8 to 31) at C7 level. Clustering results are shown on the left half of each spinal cord, while the Watson atlas is shown on the right half.

To get a sense for how the fixed number of clusters affect the results, Figure 7 shows clustering results when the number of clusters was varied from 7 to 30. This result is presented for a representative slice (C7 level). Visually, a better correspondence is observed with a higher number of clusters. More specifically, the gracile fasciculus (black) and the lateral spinal nucleus (yellow) start to appear more consistently as the number of clusters increase. However, even with a relatively high number of clusters, the rubrospinal tract (purple) is hard to distinguish. Interestingly, at 13 clusters and higher, the dorsal corticospinal tract (cyan) is horizontally split between two clusters, suggesting two different fiber populations within this tract.

## Discussion and conclusion

We aimed to obtain a topological representation of white matter tracts in the rat spinal cord using a data-driven clustering method from axon and myelin morphometric features computed from electron microscopy. In the following sections we interpret the results, discuss the limitations of the method and present some perspectives.

### Histology-driven clustering

The slice-wise clustering generated two clusters that were relatively stable across slices and that corresponded well to the topology of the Watson atlas, namely the cuneate fasciculus and the dorsal corticospinal tract (See Figure 4). Other (smaller) clusters were less consistent. We hypothesized that this lack of consistency was mainly due to the noise in the histology maps, and that more robust clustering would be obtained by aggregating more slices together (see additional considerations in the discussion below). The region-wise clustering did indeed produce clusters that were more consistent across regions, and with more clusters matching the topology of the Watson atlas. Despite that, some smaller tracts (gracile, postsynaptic dorsal column, lateral spinal nucleus, rubrospinal) were not well captured by the clustering. The ventral funiculus, which is a large collection of ventral tracts from the Watson atlas, appears parcellated on the generated clusters, and this parcellation is not consistent across slices.

This lack of consistency is possibly related to noise from the histology data and/or to the ill-posed nature of clustering (Rajapakshage and Pensky, 2020). Indeed, clustering assumes that (i) a solution exists, (ii) the solution is unique and (iii) the data provide sufficient information to reach the solution. In this project, a core assumption was that tracts could be identified from each other only based on their morphometric signature. This immediately questions the choice of morphometrics that were selected for this clustering work. Here, we included the following metrics: axon density, axon diameter, axon volume fraction, g-ratio and myelin thickness. This decision was based on the qualitative and quantitative observation of the variability of each of these metrics across spinal tracts. However, it is possible that this choice was suboptimal, and that clustering would benefit from removing some metrics or adding others. For example, the g-ratio is relatively flat across tracts, so it could potentially be removed from the analysis (hence reducing the noise). To investigate the effect of selecting less metric maps for the clustering, we rerun the slicewise clustering with only the axon density and the axon volume fraction maps (see results in Supplementary Figure 2). Interestingly, the clustering result is fairly similar to that when using all five metric maps, suggesting that some of the metrics used in the initial clustering analysis are mostly contributing. Further work could focus on the optimal choice of axon/myelin morphometrics for clustering spinal tracts.

**FIGURE 8:**
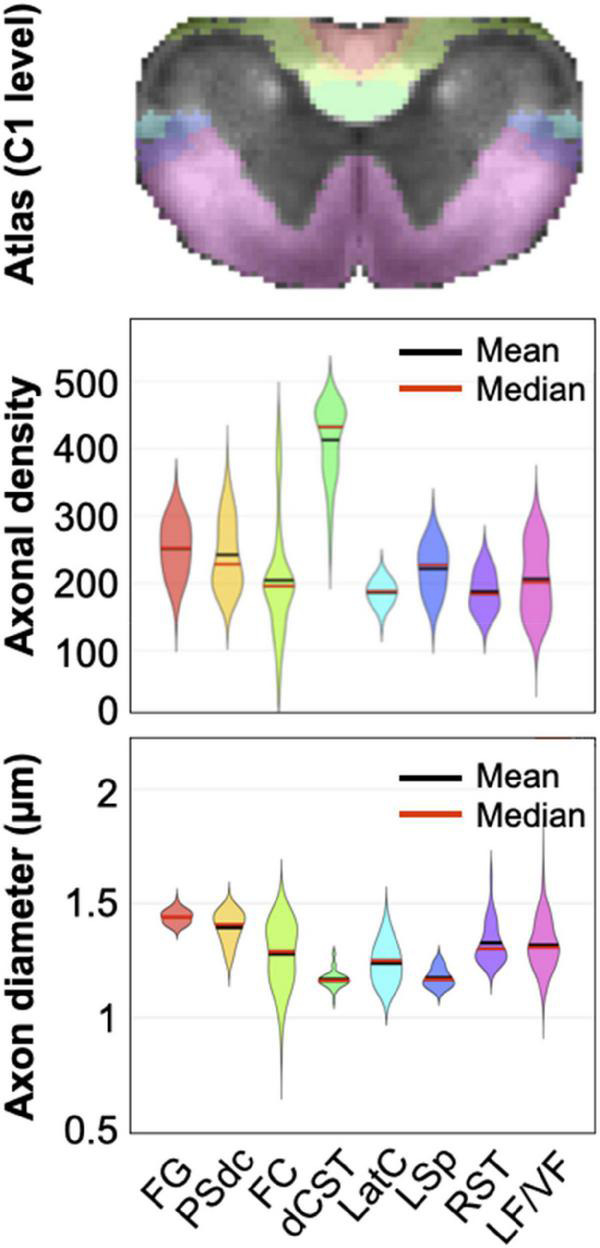
Violin-plot distribution of axonal density (number of axons per 2500 μm^2^ area) and axon diameter in the rat spinal cord at C1 level in the fasciculus gracilis (FG), the postsynaptic dorsal column (PSdc), fasciculus cuneatus (FC), dorsal corticospinal tract (dCST), lateral cervical nucleus (LatC), lateral spinal nucleus (LSp), rubrospinal tract (RST) and the lateral and ventral funiculi (LF/VF), as defined by the Watson atlas. While the metrics median (red lines) and mean (black lines) vary across tracts, their distribution clearly overlap. Modified with permission from Saliani et al. (2019).

Another (strong) assumption is that each tract is considered ‘homogeneous’, i.e., the axons that compose them are assumed to all have the same morphometrics (e.g., axon diameter of 800 nm, myelin thickness of 500 nm). We know very well this assumption is wrong, because axons that compose a given white matter tract exhibit a relatively wide range of morphometrics (Saliani et al., 2017, 2019; Duval et al., 2019). For example, Saliani et al. (2019) showed that the distributions of axonal density clearly overlap across most tracts, as illustrated in Figure 8. Interestingly, the dorsal corticospinal tract has a very different distribution from the other tracts (much denser axons), which likely explains the relatively good stability of that cluster in the present study. Similarly, axon diameter distributions also overlap across multiple tracts. The implication, from a clustering point-of-view, is that it makes the morphometric-based separation of individual tracts particularly challenging, because of the ill-posed nature of the problem.

The decision to perform region-wise clustering was twofold. Firstly, without the region-wise aggregation, clustering results appeared somewhat noisy and unreliable across adjacent slices, hence motivating the aggregation of pixels sharing similar anatomical features, even though we acknowledge this may be an oversimplification in some parts of the anatomy, especially in small tracts which shape and location is changing along the rostro-caudal axis. The medium and bigger tracts, however, show some levels of spatial overlap within a given region as can be qualitatively observed in the Watson atlas. Secondly, it has been quite common in the neuroanatomy community to represent the topology of the spinal cord white matter within the cervical, thoracic, lumbar and sacral. For example, the Gray’s anatomy atlas of the human spinal cord only shows one level per region (Standring, 2008). The work of Nathan et al. (1990), which focuses on the corticospinal tract in humans, does report that “*the arrangement [of the lateral CST] is more constant throughout the thoracic segments*,” although, to be fair, more topological variability is observed within the cervical spinal cord (notably around the cervical enlargement). The idea behind using non-linear bspline-regularized registration based on the shape of the gray matter and spinal cord, however, is somewhat supported, in the same article by Nathan et al., by the observation that the “*location of the lateral corticospinal tract is influenced by the size and position of the anterior and posterior horns as well as by the shape of the cord itself*.” However, this observation only concerns the CST, and other tracts might be displaced differently than the CST. To sum up, while we acknowledge that the proposed within-region clustering is an oversimplification, especially for smaller tracts, the slowly varying topology of the white matter tracts along the rostro-caudal direction is not a new idea, and while the region-wise aggregation is possibly too aggressive, the proposed methodology could be adapted to apply the rostro-caudal regularization between fewer slices (e.g., 3–5 adjacent slices, instead of within a full region).

The clustering algorithm asked for a fixed number of clusters to be generated. We started with 8, based on the number of tracts identified in the Watson atlas. Increasing the number of clusters (up to 30) produced better delineation of some tracts that were otherwise not captured with a smaller number of clusters. This is not surprising, given that more clusters enable adjacent regions with subtle morphometric differences to be separated. However, fixing a high number of clusters also defeats the purpose of automatically finding one and only one cluster associated with an individual tract.

The previous considerations naturally bring up the question of validation. As mentioned in the methods section, a quantitative validation of the clustering results would have been convoluted due to the exploratory nature of the present work. For example, computing a spatial similarity metric between the clusters and tracts from the Watson atlas would be inherently biased toward the arbitrary pairing between a given cluster and a given tract. As we observed, some tracts are represented by several clusters (especially in the ventral funiculi), therefore it is unclear how to best compute a spatial similarity in those instances. Instead, we opted for a visual interpretation of the produced clusters, aided by a color-coding and intensity leveling computed from the overlap between the produced clusters and each of the tracts from the Watson atlas.

Future work could also explore inputting spatial prior to the clustering algorithm for making the clusters more stable and reliable. For example, spatial priors based on the existing Watson atlas (and/or other atlases) would enforce the identification of tracts that are difficult to identify solely based on their morphometrics.

### Histology technique

There exist different histology techniques with their pros and cons. Optical microscopy can provide a full coverage of the slices but at a limited resolution (∼200 nm). The other extreme, transmit electron microscopy, can provide nanometric resolution, but only on small spatial windows (about 50 μm × 50 μm) taken sparsely across the tissue, hence it does not provide a full picture. Blockface transmit electron microscopy can mitigate this covering issue, but at the cost of a more complex and expensive imaging setup. Scanning electron microscopy falls in the middle, with the possibility to achieve 50–100 nm resolution while covering the entire tissue. In this project we opted for scanning electron microscopy. While we were able to obtain morphometric maps of the entire spinal cord cross section across all spinal levels, the resolution was somewhat limiting in our ability to precisely and accurately delineate axon and myelin tissue, biasing quantities such as myelin thickness and g-ratio (Saliani et al., 2019). Also, it is very likely that very small myelinated axons (with internal diameter smaller than 200–300 nm) were missed by the segmentation algorithm and hence not accounted for in all metrics. Moreover, histology sections can be hampered by all sorts of artifacts such as improper fixation (inducing degradation of the myelin sheath), poor penetration of osmium, improper polishing, intensity bias across scanning electron microscopy sub-images and bad focus (Cohen-Adad, 2018).

### Image processing

The segmentation of individual axons and surrounding myelin sheath originated from a previous study, which relied on the AxonSeg software (Zaimi et al., 2016). We acknowledge the limitation of this fully automated histology segmentation tool, where a non-negligible amount of axons were poorly segmented (false negatives, false positives, “leaky” segmentation or sub-segmentation). However, the number of segmented instances is very high: with about 250 axons per 2500 μm^2^ (see Figure 8), and about 50,000 voxels in the white matter of the rat-atlas (Saliani et al., 2019), it sums up to 125,000,000 segmented axons and the same amount of segmented myelin, which realistically cannot be manually verified/corrected. While the AxonSeg software has since been superseded by more performant methods based on deep learning (Zaimi et al., 2018), every fully automatic has some levels of failures that will unfortunately bias the produced morphometric maps.

In addition to segmentation issues, other processing steps could have introduced undesired bias. Notably, the various filtering, inter-rat co-registration, downsampling to 50 μm resolution, and pixel-wise metric aggregation methods performed when building the metric maps which is described in Saliani et al. (2019), the co-registration between adjacent slices for the within-region clustering, and the right-left symmetrization to maximize shared information during clustering. In particular, the choice of 50 μm resolution for the metric maps (Saliani et al., 2019) results in a compromise between having sufficient axon count to obtain a reliable axon and myelin volume fraction estimates, and having sufficiently small pixel sizes to be able to resolve fine anatomical features. In the context of this clustering work, where the goal was to retrieve white matter bundles that are at least several hundreds of μm^2^ (see Saliani et al., 2017 for a review on axon morphometry in the rat spinal cord), this working resolution was likely sufficient. However, for other work looking at subtle variations within bundles, finer resolution might be desirable.

### Watson rat atlas

The ground truth used to validate the clustering results was the rat atlas of the spinal cord (Watson et al., 2009) (a.k.a. the “Watson” atlas), which is not without flaws. Firstly, this atlas was created from a single rat, hence does not represent the possible inter-specimen variability. Secondly, the white matter tracts were identified and manually drawn, based on multiple evidence from previous studies that relied on cellular injection of staining agents (Kayalioglu, 2009; Watson and Harvey, 2009). Moreover, some tracts are missing, as stated by the authors:

“*We were not able to identify the majority of long tracts in these spinal cord sections. However, their presumed position can be located with reference to Chapters 10, 11, and 12. The tracts we were able to identify were the dorsolateral fasciculus, the gracile fasciculus, the cuneate fasciculus, the postsynaptic dorsal column pathway, the rubrospinal tract, and the dorsal corticospinal tract*.” (Watson et al., 2009).

The “missing” tracts from the Watson atlas include the ventral and lateral spinothalamic tracts and the spinocerebellar tracts (ascending), the medial and lateral vestibulospinal tracts and the reticulospinal tract (descending). It is possible that our clustering method did identify some of these tracts (or at least in part), although without a proper ground truth one can only speculate. Another – possibly more interesting – approach, would be to turn the problem around, and instead of aiming at validating the clustering method based on hard-to-obtain cellular tracking techniques, the idea would be to expand our knowledge of the white matter tracts distribution by *combining* the traditional with the cluster-based techniques. There is an undeniable lack of knowledge about how white matter tracts are organized and distributed in the spinal cord across species. Including histology-driven clustering is potentially a useful technique to increase this knowledge. The open-source analysis pipeline published with this study could be useful to further develop and study white matter anatomy in rats and other species. Although this pipeline was applied to microscopic images, it can equally be used on other imaging modalities, including MRI, which offers quantitative measures of axons and myelin (Seiberlich et al., 2020).

## Conclusion

Axon/myelin morphometrics computed from histology of white matter tissue provide sufficient information to automatically identify some white matter pathways in the spinal cord. The identified tracts correspond to those where axons exhibit *very different* morphometric features compared to other tracts. However, not all tracts were correctly identified. An inherent limitation to the histology-informed clustering of white matter tracts is that the distribution of morphometrics overlaps between tracts. In other words, a ‘tract’ as neuroscientists commonly referred to (i.e., a spatially defined bundle of axons that originate or end at specific nuclei pools in the central nervous system), can be composed of multiple axon populations (e.g., small and large axons) that share common traits across tracts, making it thus challenging to retrieve their delineation solely based on the axon morphometrics. Another important consideration is that a white matter tract, while traditionally defined as a convex and non-overlapping object (e.g., an isolated “blob” in a trans-sectional slice), is not that convex and isolated: tracts in fact overlap with each other, and axons from two different tracts can cross each other, as commonly seen in the brain (Jeurissen et al., 2019). Despite these limitations, a coarse delineation of spinal tracts is useful, for example when interpreting clinical symptoms based on abnormal white matter appearance on MRI scans. Future developments of microstructure quantitative MRI even bring hope for a *personalized* clustering of white matter tracts in each individual patient.

## Data availability statement

Publicly available datasets were analyzed in this study. This data can be found here: Open Science Framework (OSF), https://osf.io/g7kx8.

## Ethics statement

The animal study was reviewed and approved by Animal Research Ethics Committee of the Montreal Heart Institute.

## Author contributions

All authors listed have made a substantial, direct, and intellectual contribution to the work, and approved it for publication.
