# Supplementary material for: Histology-informed automatic parcellation of white matter tracts in the rat spinal cord
Source: Front Neuroanat. 2022 Nov 29;16:960475. doi: 10.3389/fnana.2022.960475 (PMC9744754; doi:10.3389/fnana.2022.960475)
Supplement: Supplementary file 1 [file Data_Sheet_1.PDF]

Supplementary material

Tissue preparation is a delicate process that could hamper the quality of EM imaging, and bias subsequent quantitative analysis. Notably, osmium tetroxide, used for staining myelin, cannot penetrate tissue very well and could sometimes leave the center area improperly stained. To overcome this issue, osmium staining was performed on each cut sample (instead of the whole cord). That way, osmium penetrated both axial extremities of the sample equally along the axial plane. Following dehydration and embedding, the polishing step removed only a few 10-100 microns of tissue, which osmium properly penetrated. **Figure S1** shows an overview SEM image for 6 rats to appreciate the homogeneity of osmium staining across the entire axial view.

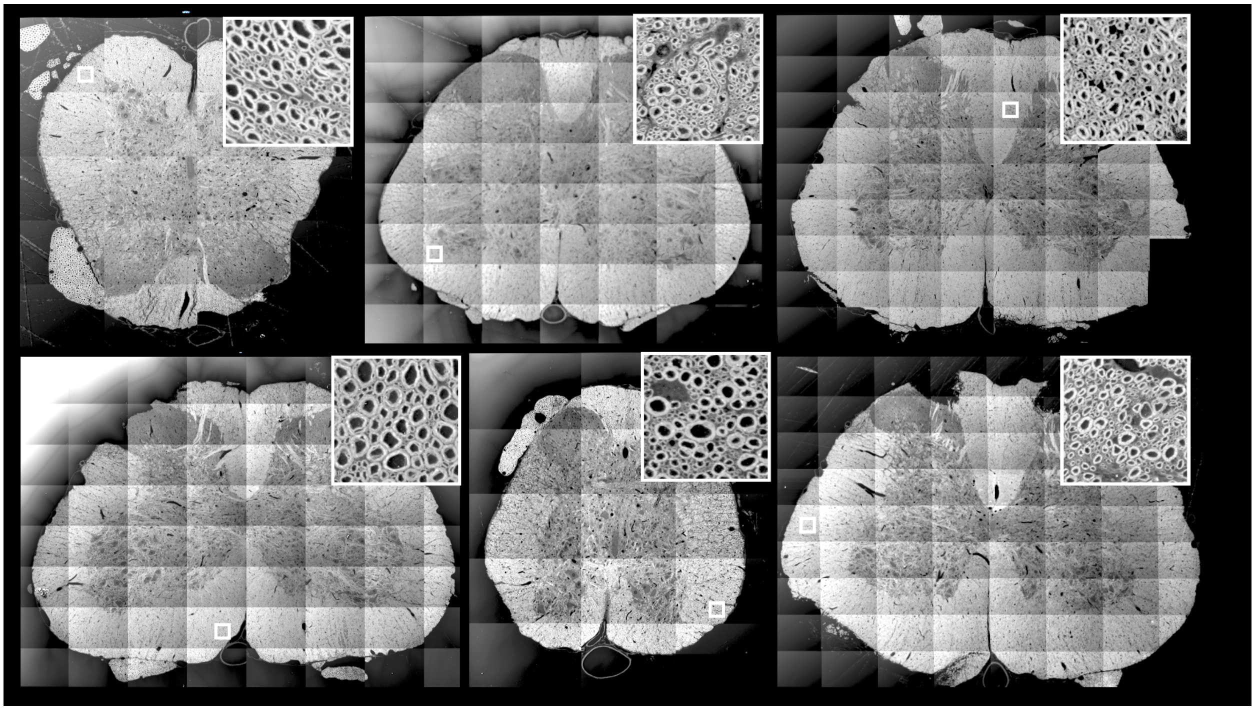

Figure S1. Stitched SEM images in six different rats, five of which were used to produce the metric maps. On each view, one can observe the smaller tiles used for a single SEM acquisition. Each tile is subjected to intensity inhomogeneity, hence the ‘checkerboard’ pattern when stitching all tiles together. The purpose of this figure is to appreciate the homogeneity of osmium staining: all parts of the spinal cord, especially at the center, are properly stained with osmium (ie: there is no visible signal drop out). On each of the six SEM images, an inset shows a higher magnification to appreciate the resolution and contrast of myelinated axons.

To investigate the effect of selecting less metric maps for the clustering, we rerun the slicewise clustering with only the axon density and the axon volume fraction maps. These two maps were chosen as they appear to have the most tract-specific signatures, while the other maps might mostly contribute to noise in the clustering algorithms. Results are shown in **Figure S2**. Interestingly, the clustering result is fairly similar to that when using all five metric maps, suggesting that some or all of the remaining metrics (axon diameter, g-ratio, myelin thickness) do not contain sufficient tract-specific information for parcellation.

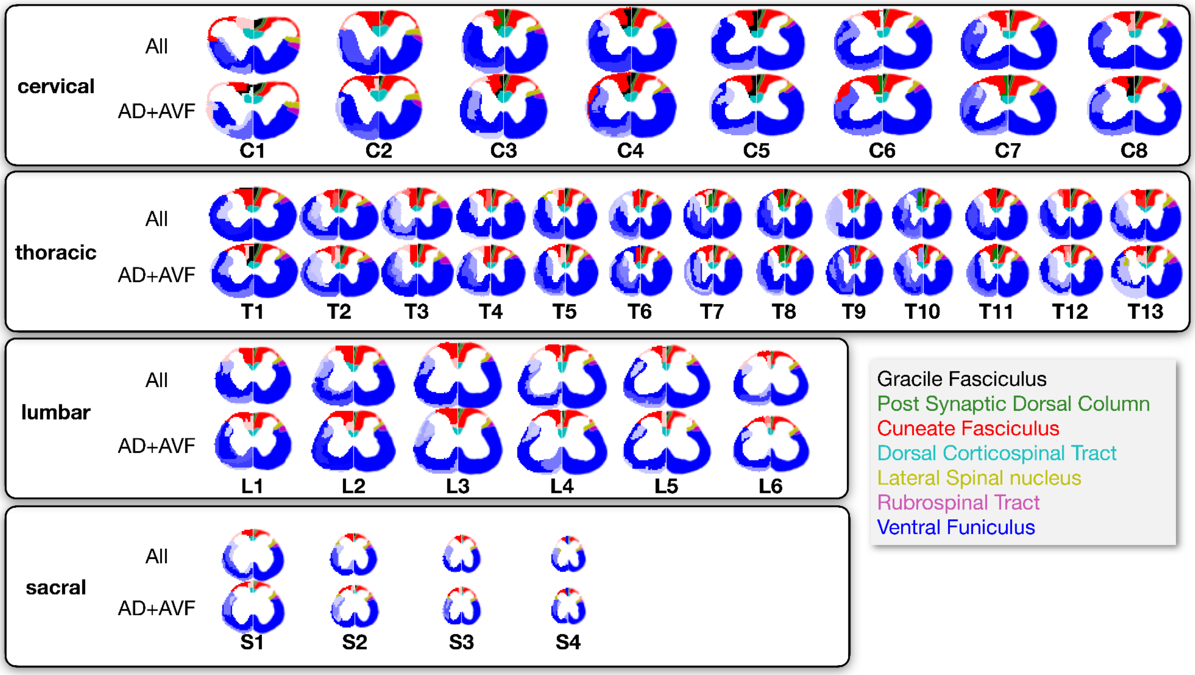

Figure S2. Results of the slice-wise clustering for each spinal level (set to 8 clusters), and using either all five metrics (as in **Figure 4**), or only the axon density and axon volume fraction. Clustering results are shown on the left side of each spinal cord, and the Watson atlas is shown on the right side.
